# Graphene‐Enhanced Plasmonic Interfaces: A General Strategy for Highly Sensitive Detection of Biomolecular Interactions

**DOI:** 10.1002/adhm.202501723

**Published:** 2025-09-04

**Authors:** Ahmar Hasnain, Heiko Heilmann, Nghi Luong Phuong Le, Klaus‐Ingmar Pfrepper, Andreas Wruck, Peter Groß, Bernd Bufe, Alexey Tarasov

**Affiliations:** ^1^ Faculty of Computer Sciences and Microsystems Technology Kaiserslautern University of Applied Sciences Amerikastr. 1 66482 Zweibrücken Germany; ^2^ Faculty of Applied Logistics and Polymer Sciences Kaiserslautern University of Applied Sciences Carl‐Schurz‐Str. 10‐16 66953 Pirmasens Germany; ^3^ PROGEN Biotechnik GmbH Maaßstraße 30 69123 Heidelberg Germany

**Keywords:** biosensor, cells, monolayer graphene, surface plasmon resonance (SPR), virus

## Abstract

The detection of cells and viruses is essential for research and clinical applications, creating a demand for high‐performance biosensors. Surface plasmon resonance (SPR) enables label‐free, real‐time detection and is highly promising for healthcare, including point‐of‐care diagnostics. However, its performance is often limited in complex biological systems. Integrating two‐dimensional (2D) materials such as graphene into SPR sensors has been proposed as a strategy to improve sensitivity, but experimental evidence remains scarce. Here, we investigate the influence of graphene on SPR biosensors using several relevant biological examples, including antibody‐virus and peptide‐cell interactions. Compared to gold sensors, graphene integration produced reproducible signal enhancement of up to 600%, far exceeding previous reports.  Importantly, graphene‐enhanced SPR enabled discrimination between different cell types, a capability not observed with gold alone. Our findings demonstrate that graphene provides substantially greater enhancement than predicted and can be applied across diverse biological systems. This establishes graphene‐enhanced SPR as a powerful platform for advancing biosensor performance, with broad potential in biomedical research, diagnostics, and gene therapy.

## Graphical abstract

Monolayer graphene coating significantly enhances the sensing performance of conventional SPR biosensors. Its impact is evaluated using biologically relevant models, including antibody‐virus and peptide‐cell interactions. Graphene‐enhanced SPR enabled specific virus detection via antibodies and effective cell type discrimination using synthetic peptides, capabilities that surpass those of traditional gold‐based SPR sensors.

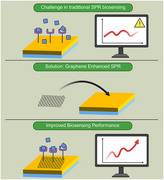

## 1 Introduction

The detection of cells and viruses is critical for both life science research and healthcare applications. For example, white blood cells (leukocytes) are an important part of the immune system. Infections, leukemia, and other diseases affect the number and function of different types of leukocytes in the blood. Changes in their composition and biological activity are therefore important markers of health status. Another relevant example is the specific detection of viruses, which plays an important role in laboratory diagnostics as well as in pharmaceutical research, including the development of virus‐based gene therapies and vaccines.

The most common traditional biological detection methods include the visualization of cells via microscopy,^[^ ^1^ ^]^ flow cytometry for analyzing cell characteristics,^[^ ^2^ ^]^ immunohistochemistry (IHC) for detecting specific markers in tissues,^[^ ^3^ ^]^ PCR‐based methods for identifying cells,^[^ ^4^ ^]^ and ELISA for detection and quantification of antigens, antibodies, or proteins.^[^ ^5^ ^]^ However, these standard methods have various limitations, such as time‐consuming pre‐treatment procedures, labeling, stability, renewability, cross‐reactivity, and the requirement of trained professionals for certain methods. Different alternative label‐free sensing methods, such as electrochemical sensors,^[^ ^6^, ^7^, ^8^ ^]^ microcantilever arrays,^[^ ^9^ ^]^ plasmonic fiber sensors^[^ ^9^ ^]^ and surface plasmon resonance (SPR) sensors are being developed^[^ ^10^, ^11^, ^12^, ^13^, ^14^ ^]^ to avoid these constraints in classical detection methods. Unlike many other immunoassays, SPR biosensors are label‐free, fast, and allow real‐time monitoring of biological interactions with many potential healthcare applications, including point‐of‐care diagnosis.

**Figure 1:**
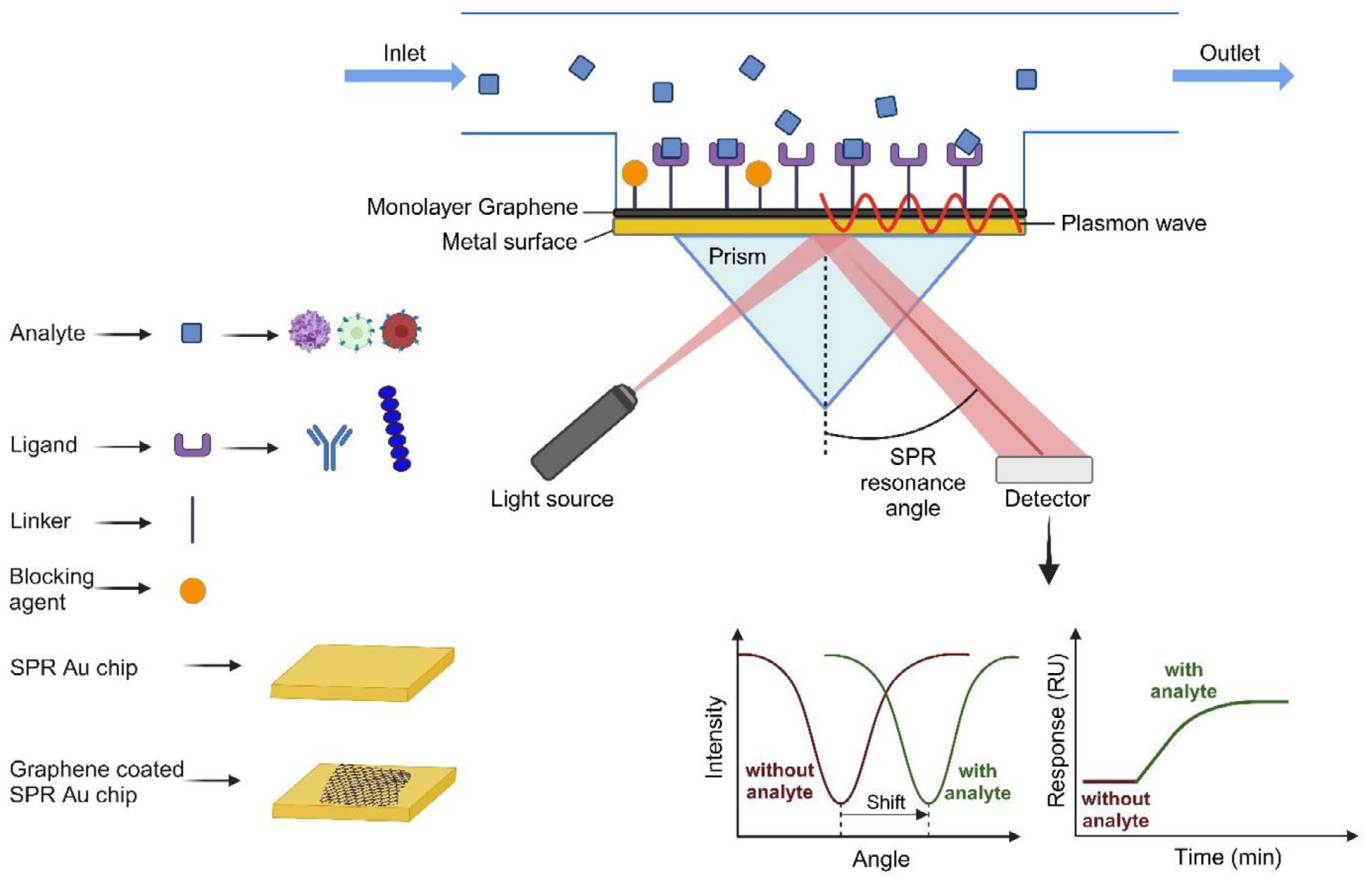
Schematic of Graphene‐Enhanced Surface Plasmon Resonance (SPR) equipped with a glass prism in Kretschmann configuration, and the detection assay used for this study.

A schematic of an SPR immunoassay is shown in **Figure** **1**. Capture molecules (ligands, e.g., antibodies or short peptides) are immobilized on the surface of the SPR chips. The analytes (e.g., virus or cell) are in solution and can interact with immobilized ligands, which are monitored in real time without any label molecules. The cell‐based SPR approach also offers a more accurate representation of cellular behavior, closely mimicking actual biological systems, thereby providing a more realistic analysis of cellular interactions.^[^ ^15^ ^]^ For the real‐time detection, SPR senses variations in the refractive index near a metal surface. When incident light strikes the metal layer at a specific angle, it induces the excitation of surface plasmon‐electron oscillations that are extremely sensitive to variations in the surrounding medium.^[^ ^16^ ^]^ Binding events between ligands and analytes result in a resonance angle shift that allows for precise and continuous measurement of these interactions. Thus, this shift also depends on the refractive index changes of the medium.^[^ ^17^ ^]^

For the reasons described above, SPR is especially valuable for biomedical research and applications, yet performance limitations remain.^[^ ^18^ ^]^ Initial studies suggest that the performance of SPR sensors can be further enhanced by using 2D materials such as graphene,^[^ ^19^, ^20^, ^21^, ^22^, ^23^ ^]^ induced by their optical properties and minimal thickness.^[^ ^24^ ^]^ This enhanced sensitivity can enable real‐time detection and characterization of molecular interactions, critical for biosensing applications. Additionally, graphene's high surface‐to‐volume ratio facilitates the increased and optimized immobilization of linker and ligand molecules on the sensor surface, thereby improving sensor performance.^[^ ^25^, ^26^ ^]^ The biocompatibility of graphene further underscores its suitability for biosensing applications. While monolayer graphene has been extensively utilized in electrochemical biosensors,^[^ ^27^, ^28^, ^29^, ^30^ ^]^ its application in enhancing SPR sensor sensitivity is not well studied. In particular, graphene‐enhanced SPR sensors have not been used to study interactions between living cells and peptides nor between viruses and antibodies, both highly relevant biological systems with many potential healthcare applications. Also, it is not clear to what extent the signals can be enhanced experimentally.

Theoretical studies showed that the strong plasmonic coupling between gold and graphene facilitates efficient plasmon propagation and enhances the interaction between the two materials.^[^ ^31^ ^]^ This effect leads to a sensitivity enhancement that depends on the number of graphene layers, with a maximum enhancement when 10 layers of graphene are used.^[^ ^17^, ^20^, ^32^ ^]^ It is difficult to achieve experimentally a homogeneous and exact number of CVD‐grown graphene layers, which affects the reproducibility of SPR sensors coated with few layers of graphene. Other studies have also looked at hybrid interfaces, combining graphene with other 2D materials such as MoS_2_ and PtSe_2_,^[^ ^33^, ^34^ ^]^ which increase the complexity of the system.

In this study, we focus on a relatively simple approach by integrating monolayer graphene to improve the performance of surface plasmon resonance (SPR) sensors, as shown in Figure 1. We demonstrate the fabrication of a highly sensitive SPR sensor using high‐quality monolayer graphene, grown by chemical vapor deposition (CVD) and transferred to SPR chips. Our results show that SPR chips coated with monolayer graphene achieved significantly higher signals due to antibody immobilization and virus binding compared to standard gold chips. We also investigated peptide‐cell interactions, where graphene‐coated chips showed superior peptide immobilization compared to traditional gold chips. Using peptide‐coated graphene, we were able to effectively differentiate between two immune cell line models, which was not possible with gold alone. In all biological systems studied, graphene consistently enhanced the signals (up to 600%), far exceeding enhancement factors reported in previous theoretical and experimental work. These results highlight the potential of graphene to significantly advance label‐free and real‐time biosensors for various healthcare applications.

## 2 Results and Discussion

### 2.1 Virus Sensing on Gold and Graphene Enhanced SPR Sensors

Adeno‐associated virus (AAV) is one of the vectors mostly used for gene therapy. Several monoclonal antibodies against AAV have been established that are used to detect AAVs in conventional assays in the context of gene therapy. Binding analysis between AAV and monoclonal as well as patient antibodies plays an important role in gene therapy research.^[^ ^35^ ^]^

**Figure 2:**
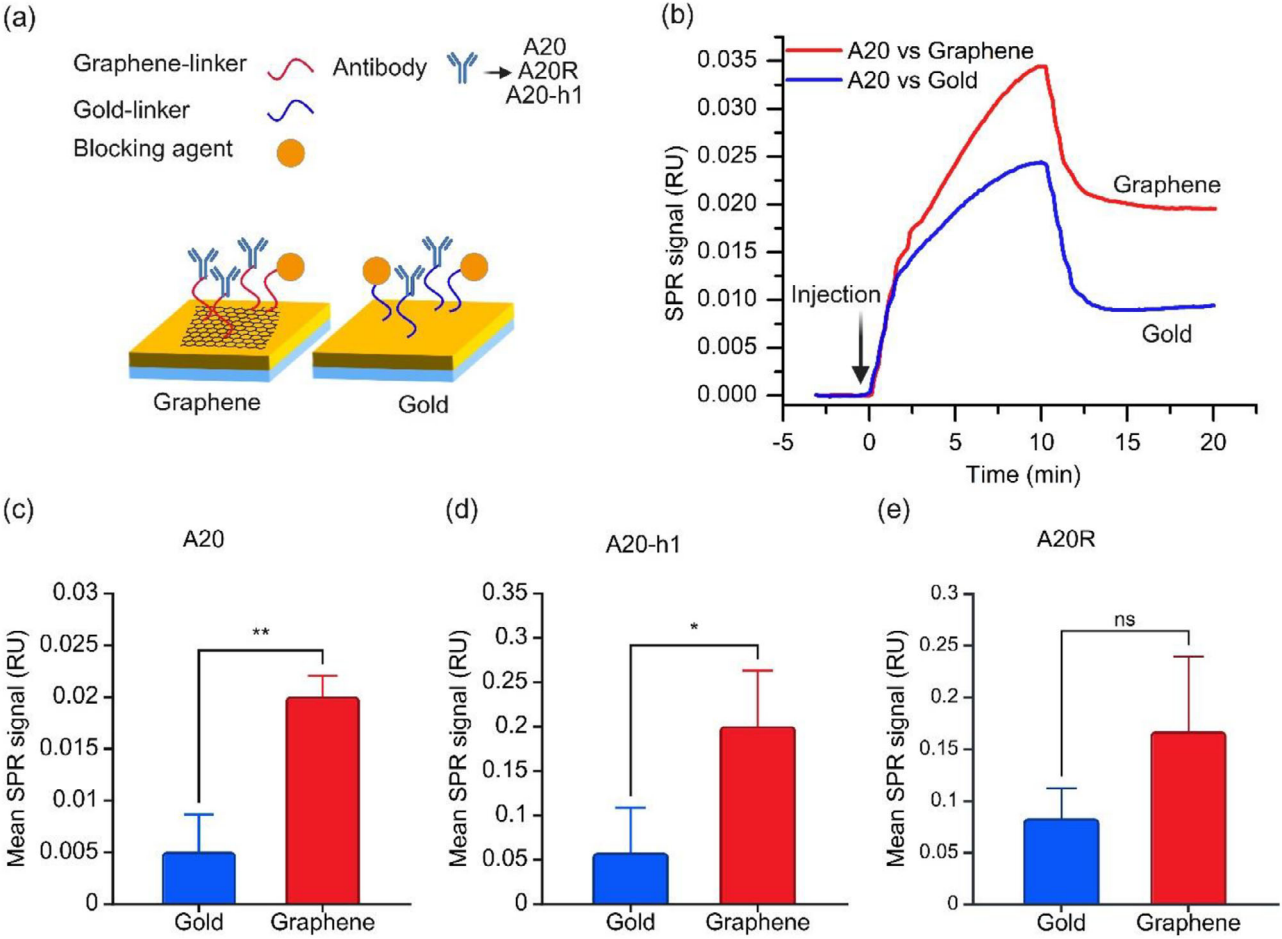
Immobilization of three different antibodies on gold and graphene using linker molecules. a) A schematic showing how antibodies that differ in their Fc region are chemically attached to gold and graphene‐coated SPR sensor chips. b) SPR sensogram showing the chemical bonding of A20 antibodies. c–e) Bar charts comparing immobilization of A20‐(c), A20‐h1‐(d), and A20R‐(e) on graphene (red) and gold (blue) chips, based on sensorgram data. Bars represent the mean ± SD (n= 3). Statistical significance between groups was determined using an independent samples *t*‐test, n=3. Significance levels are indicated as follows: ns, *p* ≥0.05; ^*^, *p* < 0.05; ^**^, *p* < 0.01; ^***^, *p* < 0.001.

First, the chemical immobilization of three monoclonal antibodies A20, A20R, and A20‐h1 was demonstrated on gold and graphene‐coated SPR sensors (schematic representation in **Figure** **2**). The antibodies were covalently attached by amine coupling to short linker molecules containing reactive butyric acid moieties (four carbon atoms). Two different linker molecules of similar length were used to modify gold and graphene: 4‐mercaptobutyric acid (4‐MBA) binds covalently to gold, and 1‐pyrenebutyric acid (PBA) binds to the graphene surface via π‐π interactions.^[^ ^36^ ^]^ The PBA provides surface carboxyl groups,^[^ ^37^, ^38^ ^]^ which are activated using EDC (1‐ethyl‐3‐(3‐dimethylaminopropyl)carbodiimide) and NHS (N‐hydroxysuccinimide) to form reactive NHS esters.^[^ ^39^ ^]^ These intermediates subsequently react with primary amine groups on lysine residues of the antibody, resulting in the formation of stable covalent amide bonds.^[^ ^40^ ^]^

After incubation with linker molecules, an antibody concentration of 8 µg ml^−1^ was injected into the SPR fluidic chamber. In Figure 2b the SPR sensogram shows successful immobilization of the A20 antibody on gold and graphene, with a clear improvement in immobilization on the graphene sensor. As shown in Figure 2c–e, all three antibodies achieved higher immobilization on the graphene surface, with A20 showing the highest enhancement (fourfold) compared to the gold surface. These results suggest that adding monolayer graphene increases signals resulting from antibody immobilization. Future work will optimize the surface modification process to improve reproducibility and reduce standard deviation. For instance, site‐specific, oriented antibody immobilization has recently been demonstrated to enhance the sensitivity and reproducibility of graphene biosensors.^[^ ^41^ ^]^ **Table** **1** describes the exact SPR signal enhancement data achieved by these antibodies on graphene compared to gold.

**Table 1:** Overview of Results Compared with Related Experimental Studies.

Capture molecule	Target molecule	Enhancement factor [%]	Refs.
biotinylated BSA	Streptavidin	37	[19]
biotinylated cholera toxin (antigen)	Cholera Toxin (antibody)	80	[24]
1‐Pyrene butyric acid	Antibody (A20)	400	This work
1‐Pyrene butyric acid	Antibody (A20‐h1)	348	This work
1‐Pyrene butyric acid	Antibody (A20R)	201	This work
A20	Virus (AAV2)	373	This work
A20‐h1	Virus (AAV2)	222	This work
A20R	Virus (AAV2)	385	This work
1‐Pyrene butyric acid	Peptide (f‐MVPIKI)	300	This work
1‐Pyrene butyric acid	Peptide (f‐MEQQNK)	601	This work
f(MVPIKI)	Cell (THP1)	221	This work
f(MVPIKI)	Cell (HL60)	183	This work
f(MEQQNK)	Cell (THP1)	119	This work
f(MEQQNK)	Cell (HL60)	134	This work

All three antibodies (A20, A20R, and A20‐h) were tested against AAV2, one of the most commonly used AAV serotypes in the gene therapy field, to compare pure gold and graphene‐coated gold chips. It is important to note that all three antibodies have different Fc regions, which may result in a different amount of antibody binding to the sensor surface. The interaction between the antibody and the virus occurs through reversible, non‐covalent forces, such as hydrogen bonding, ionic interactions, van der Waals forces, and hydrophobic effects.^[^ ^42^, ^43^, ^44^ ^]^ A20 is a mouse IgG3 monoclonal antibody that neutralizes AAV2 by binding to a conformational epitope on assembled AAV2 capsids.^[^ ^45^, ^46^, ^47^ ^]^ A20‐h1 is a recombinant human chimeric IgG1 antibody that can neutralize AAV2 and AAV3. A20R is described as a recombinant mouse IgG1 antibody that specifically interacts with AAV2, AAV3, and Anc80 particles. AAV2 is specific to this family of antibodies and therefore expected to interact with all of them, while AAV8 is used as a virus control and should show no reactivity to these monoclonal antibodies.

**Figure 3:**
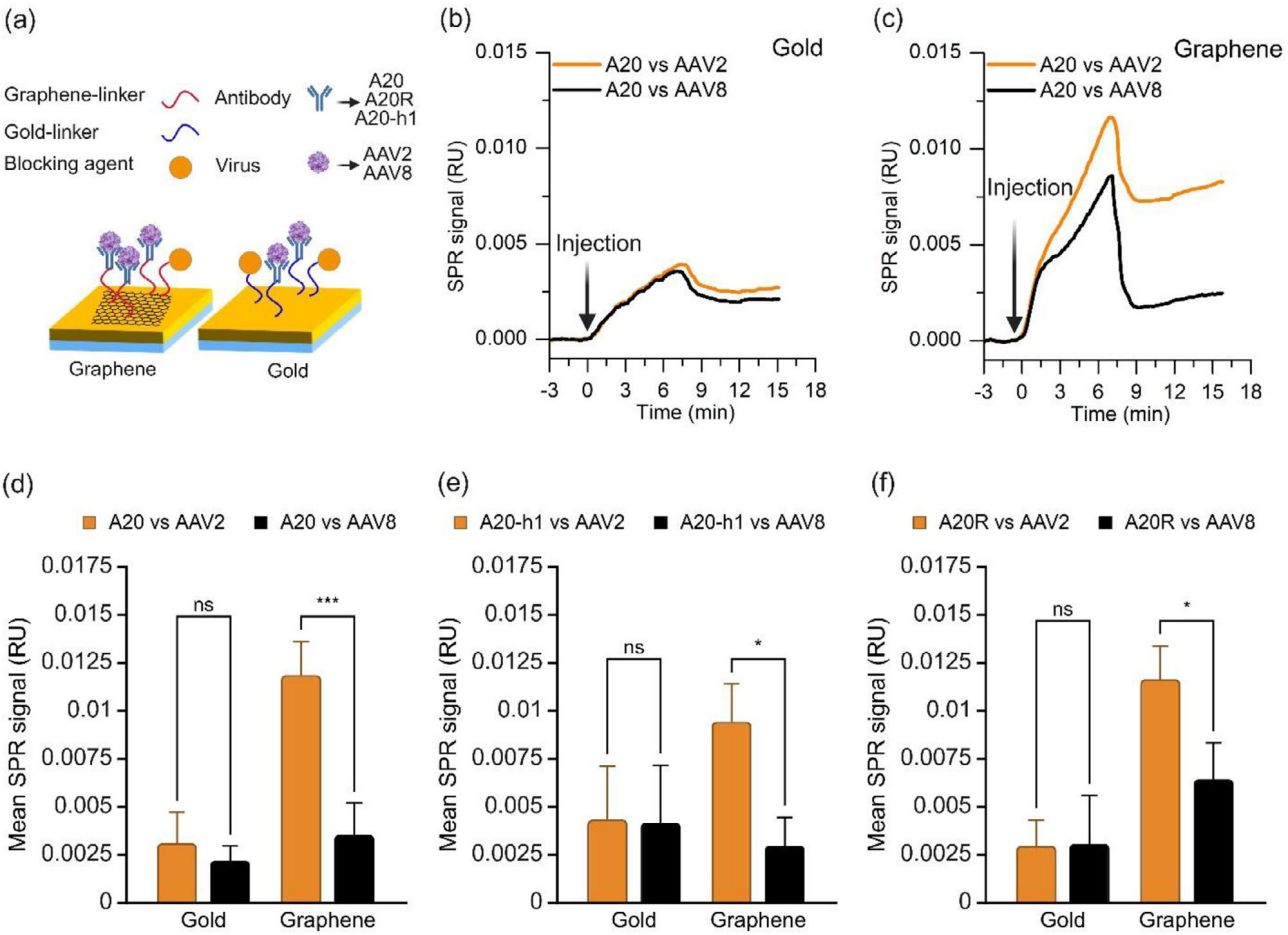
Comparison of virus particles captured on gold and graphene sensors. a) Schematic representation of detection assays to capture AAV2 on gold and graphene enhanced SPR sensors, while AAV8 is used as a virus control b) SPR sensograms showing AAV2 (orange) and AAV8 (black) interaction to immobilize antibody A20 on gold chips. c) SPR sensograms showing AAV2 (orange) and control virus AAV8 (black) interaction to immobilize antibody A20 on graphene chips. d–f) Bar charts comparing interactions of graphene and gold sensors, functionalized with three different antibodies (A20 (d), A20‐h1 (e), and A20R (f)), with AAV2 (orange) or control virus AAV8 (black), based on sensorgrams data. Bars represent mean±SD. Statistical significance between groups was determined using Two‐way ANOVA, n = 3. Significance levels are indicated as follows: ns, *p* ≥ 0.05; ^*^, *p* < 0.05; ^**^, *p* < 0.01; ^***^, *p* < 0.001.

**Figure** **3**a shows the schematic of our biosensing assay for antibody‐virus interaction, performed on gold‐coated SPR chips with and without graphene. On gold surfaces functionalized with the A20 antibody, injection of AAV2 and AAV8 produced relatively small signals, with no clear distinction between the two viruses (Figure 3b). By contrast, graphene‐based sensors functionalized with A20 (Figure 3c) showed markedly higher AAV2 signals, with AAV2 producing a stronger response than the control virus AAV8. Notably, the sensogram for A20 immobilization on the linker‐modified surface (Figure 2b, red curve) displayed a steeper slope and greater signal magnitude compared with the sensogram for AAV2 binding on the antibody‐coated surface (Figure 3c, red curve). These differences arise from the distinct interaction mechanisms: covalent immobilization of antibodies involves rapid and irreversible attachment to the surface, resulting in a steep slope and strong signal, whereas virus–antibody binding is slower, reversible, and biologically specific, leading to a shallower slope and weaker signal. In addition, the higher concentration of A20 antibody used for immobilization (8 µg/ml) compared to that of AAV2 (10 pM) further contributes to the observed signal differences.

Figure 3d–f  compares the mean SPR signal recorded upon exposure of gold and graphene surfaces, each functionalized with three different antibodies, to AAV2 (orange) and AAV8 (black). All three antibodies displayed similar behaviour. On graphene, AAV2 produced significantly larger signals than AAV8, whereas on gold no significant difference was observed between the two viruses. This is consistent with the lower signal at the detection limit^[^ ^48^ ^]^ (S_dl_ = 0.0007) of graphene compared to gold (S_dl_ = 0.0018), indicating enhanced sensitivity of the graphene‐coated SPR sensor. Furthermore, the lower baseline drift (3.7×10^−5^ RU min^−1^) highlights the stability of these sensors. To evaluate performance in complex biological samples, we tested the effect of non‐specific binding using two concentrations of bovine serum albumin (BSA): 0.0035% and 0.35% w/v (0.035 g/L and 3.5 g/L), as shown in Figure S5 (Supporting Information). Despite increased non‐specific binding, the biosensor maintained specificity, clearly detecting AAV2 binding to A20 antibodies and demonstrating functionality in protein‐rich environments.  While the tested BSA levels are lower than physiological albumin concentrations (3.5–5g/dL),^[^ ^49^, ^50^ ^]^ they reflect relevant conditions, as future applications will include sample pretreatment and dilution to reduce matrix effects^[^ ^51^ ^]^ and nonspecific adsorption.^[^ ^52^, ^53^, ^54^ ^]^

In summary, all three antibodies were able to interact significantly better on graphene sensors than on gold sensors. As shown in Table 1, the graphene surface always gave more signal at the same concentration from the immobilized antibodies. For example, up to 3.85 times more signal on graphene than on gold for AAV2 detection agrees very well with up to 4 times more antibody signal. This can be partly explained by the fact that the carboxyl group of PBA exhibits repulsive behavior toward the graphene surface and faces away from the sensor surface, improving the vertical orientation of the antibodies on the graphene surface.^[^ ^36^ ^]^ This potentially increases the density and availability of antibody binding sites for the virus particles, resulting in higher immobilization on graphene. This in turn suggests that AAV2 particles had a greater chance of interacting on graphene sensors compared to gold sensors.

### 2.2 Peptide‐Cell Interaction on Gold and Graphene Enhanced SPR Sensor

Can the significant signal enhancement due to graphene be observed in another biological system? To answer this question, we examined a peptide‐cell interaction as another relevant example. The detection of cells and the ability to distinguish between different cell types are important in biomedical research and diagnostic applications. There is a need for simple analytical tools that allow real‐time and label‐free analysis of cells. A direct chip‐based assay using immobilized capture molecules that bind cells from a biological solution is a conceptually simple way to realize such tools. While antibodies are commonly used as capture molecules, short synthetic peptides are a promising alternative due to their small size, fast and inexpensive synthesis, and high chemical stability.

**Figure 4:**
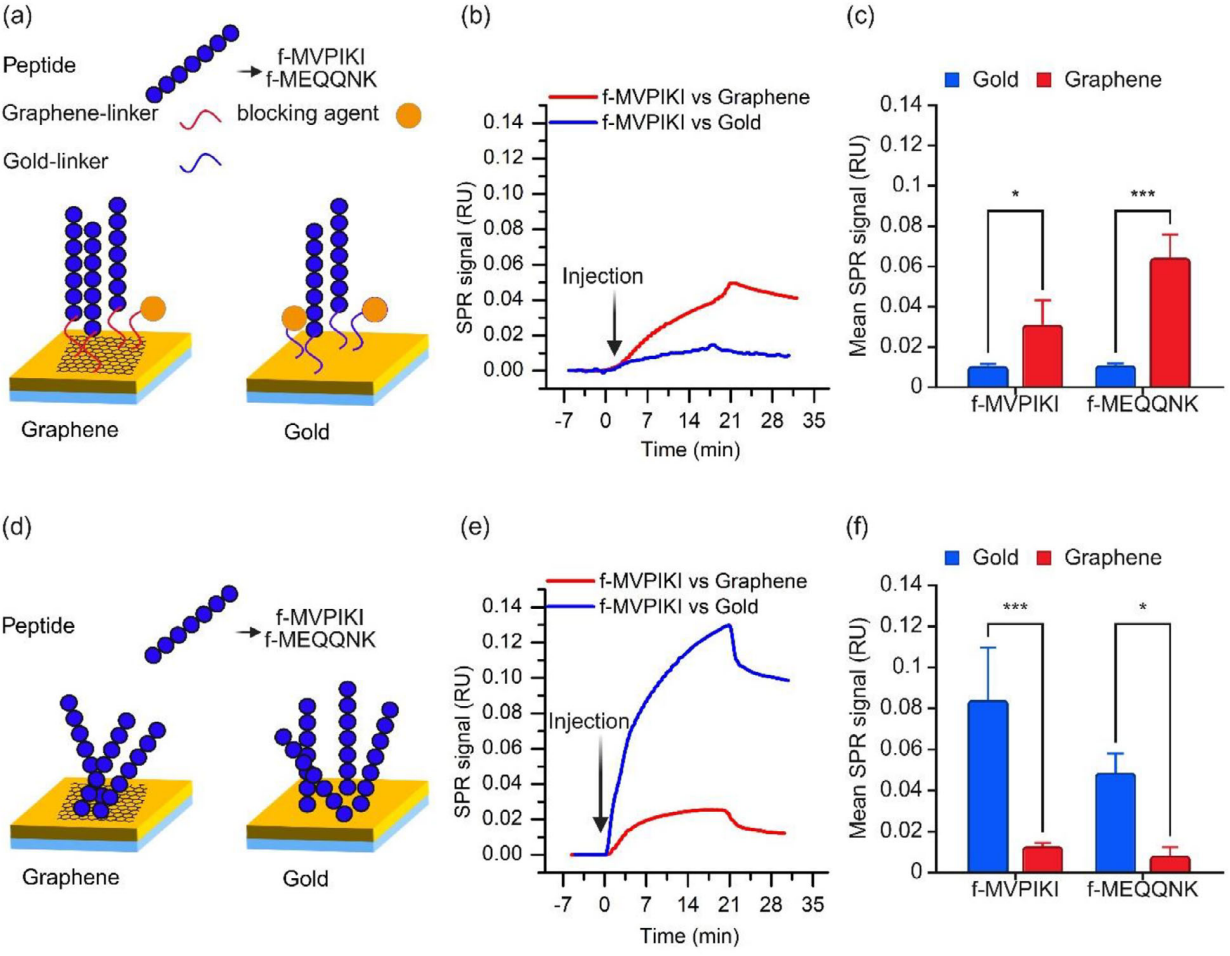
a) Schematic illustrating peptide immobilization via amine coupling chemistry on plain gold and graphene‐coated SPR sensor chips. b) SPR signal from the interaction of f‐MVPIKI with linker molecules coated on bare gold and graphene‐coated SPR sensor chips. c) Bar graph of mean SPR signals for peptide interaction with linker molecules on plain gold and graphene‐coated SPR sensor chips. d) Schematic illustrating physical adsorption of peptide on plain gold and graphene‐coated SPR sensor chips. e) SPR signal from the physical adsorption of f‐MVPIKI on bare gold and graphene‐coated SPR sensor chips. f) Bar graph of mean SPR signals for peptide interaction on bare gold and graphene‐coated SPR sensor chips. Statistical significance between groups was determined using Two‐way ANOVA, n = 3. Significance levels are indicated as follows: ns, *p* ≥ 0.05; ^*^, *p* < 0.05; ^**^, *p* < 0.01; ^***^, *p* < 0.001.

Here, we have synthesized two peptides with different cell interaction properties (f‐MVPIKI and f‐MEQQNK), which in principle can be used to distinguish between different cell types. While f‐MVPIKI is hydrophobic and has a net positive charge of +1, f‐MEQQNK is hydrophilic and has a net negative charge of −1. First, we compare the immobilization of these peptides via linker molecules on gold and graphene, see **Figure** **4**a–c.

To immobilize the peptides on the surface, the same linker molecules were used as for the antibodies in the previous section, i.e., 4‐mercaptobutyric acid (4‐MBA) for gold and 1‐pyrenebutyric acid (PBA) for graphene (Figure 4a). Since both peptides contain a lysine (K), the butyric acid can bind the peptide using the same amine coupling approach as for the antibodies. Figure 4b shows sample traces corresponding to the immobilization of f‐MVPIKI on gold (blue) and graphene (red) using amine coupling. The resulting SPR signal on graphene is much higher than that on gold. Similar data were obtained for the other peptide (f‐MEQQNK). Figure 4c summarizes these data and shows that a significantly higher response was measured on graphene than on gold for both peptides. This effect is more pronounced for f‐MEQQNK (6.1‐fold) than for f‐MVPIKI (2.2‐fold). In f‐MEQQNK, lysine (K) is the last amino acid, which may improve its availability for binding to the linker molecules. For f‐MVPIKI, lysine (K) is the second to last amino acid, which may decrease the availability for binding compared to the other peptide.

To confirm the quality of peptide immobilization a “linker control” was performed. Here, both peptides were injected over bare gold and graphene‐coated chips in the absence of linker molecules and corresponding amine chemistry as shown in schematic Figure 4d. The sensograms in Figure 4e show a high level of physical adsorption of f‐MVPIKI on gold compared to graphene. Similar results were observed with f‐MEQQNK as can be seen in Figure 4f. The interaction of f‐MVPIKI with graphene is driven by hydrophobic forces, with electrostatic interactions also playing a role. The adsorption of hydrophobic peptides onto the graphene surface is stable and can be influenced by factors such as pH.^[^ ^55^ ^]^ The peptides can also preferentially bind to the edges or planar surfaces of graphene, affecting its electronic properties.^[^ ^56^ ^]^ Due to its hydrophilic nature, f‐MEQQNK exhibits relatively low physical binding to the graphene surface, as shown in Figure 4f. Hence, hydrophobic peptides tend to absorb onto gold surfaces because they interact favorably with them. These interactions are driven by van der Waals forces and hydrophobic interactions, as well as possible chemical bonding between sulfur‐containing amino acids and gold.^[^ ^57^ ^]^ Since both peptides contain a sulfur group in the amino acid methionine, they bind more strongly to the gold surface than to the graphene surface. Conversely, hydrophilic peptides may not exhibit strong interactions with gold surfaces based solely on hydrophobic forces. However, other mechanisms, such as electrostatic interactions, can facilitate their adsorption.^[^ ^58^ ^]^ This may explain why we observe f‐MEQQNK binding less than f‐MVPIKI to the gold surface. Overall, the signal enhancement on graphene versus gold is consistent with the data obtained with antibodies, suggesting a general positive effect associated with graphene coated with linker molecules.

### 2.3 Real‐Time Monitoring of Peptide Interaction with Different Cells on Graphene and Gold Sensors

Next, we wanted to establish whether peptide‐coated sensor chips could be useful for biomedical applications. Assessing the levels of different types of immune cells in human blood samples is an important test used to diagnose a wide range of medical conditions, from infections and allergies to certain types of cancer.

**Figure 5:**
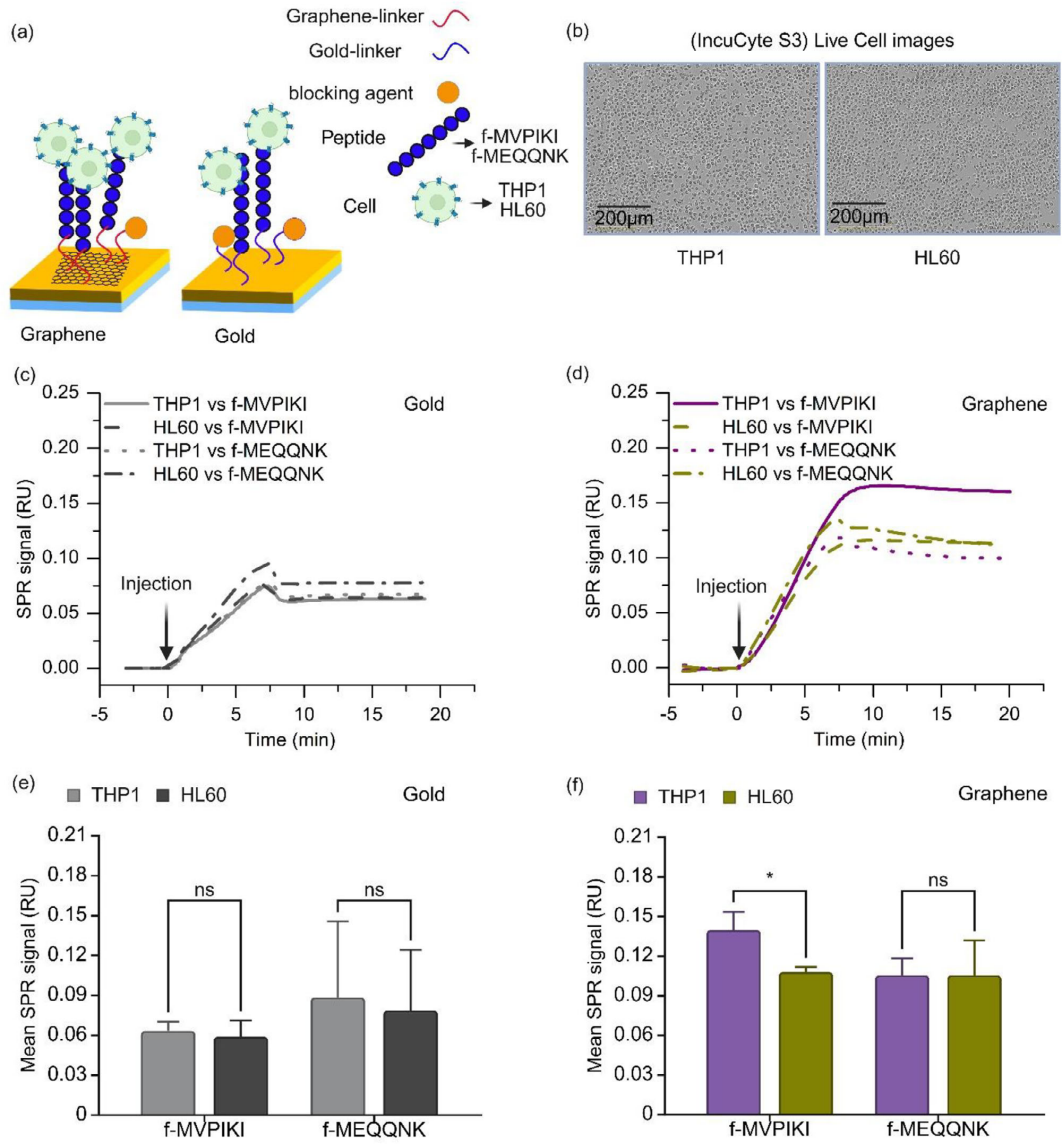
Peptide and cell interaction system. a) Schematic of peptide immobilization and subsequent capture of THP1 and HL60 cells on graphene‐coated SPR sensor chips functionalized with f‐MVPIKI or f‐MEQQNK. b) Microscopic images of THP1 and HL60 cells. c) SPR sensograms showing responses of THP1 and HL60 cells flowing over gold SPR sensor chips functionalized with f‐MVPIKI or f‐MEQQNK at 30 µl min^−1^. d) SPR sensograms showing responses of THP1 and HL60 cells flowing over graphene SPR sensor chips functionalized with f‐MVPIKI or f‐MEQQNK at 30 µl min^−1^. e) Bar graph comparing interactions of THP1 and HL60 cells with gold chips functionalized with f‐MVPIKI (navy blue) or f‐MEQQNK (dark yellow). f) Bar graph comparing interactions of THP1 and HL60 cells with graphene chips functionalized with f‐MVPIKI (dark grey) or f‐MEQQNK (light grey). Bars represent mean±SD, with n = 3 for gold and n = 4 for graphene. Statistical significance between groups was determined using a regular two‐way ANOVA. Significance levels are indicated as follows: ns, *p* ≥ 0.05; ^*^, *p* < 0.05; ^**^, *p* < 0.01.

We therefore decided to test whether the peptide‐coated sensor chips could discriminate between two relatively closely related immune cell types and how graphene or gold surfaces affected these measurements (**Figure** **5**a). Monocytes and neutrophils are the most common immune cell types in human blood samples. We therefore chose to use the leukemia cell lines THP1 and HL60, which are similar to monocytes and neutrophils, respectively (Figure 5b). Acute promyelocytic leukemia (APML) is a subtype of acute monocytic leukemia (AML) in which immature cells accumulate in the bone marrow, resulting in a reduced number of blood cells in the body. HL60 cells are derived from APML patients, while THP1 cells are derived from AML patients, but both cell types share similar expression patterns.^[^ ^59^ ^]^

This makes it difficult to distinguish between the two cell types using conventional methods. We tested the interaction of both cell types with gold and graphene sensors that were functionalized with f‐MVPIKI and f‐MEQQNK peptides. Figure 5c shows a typical SPR sensogram illustrating the interaction of THP1 and HL60 cells with a gold sensor functionalized with f‐MVPIKI and f‐MEQQNK. The observed signals are relatively low, and the different cell types cannot be clearly distinguished using peptide‐coated standard gold chips. When graphene is introduced, the signals are generally higher (Figure 5d). Moreover, there is a clear signal difference between THP1 cells and HL60 cells on graphene coated with f‐MVPIKI. These measurements were repeated with several sensors, the results are summarized as bar graphs for gold (Figure 5e) and graphene (Figure 5f).

No significant signal differences were observed between THP1 and HL60 cells on gold sensors coated with f‐MVPIKI or f‐MEQQNK. In contrast, significant signal differences were observed between THP1 and HL60 cells on graphene‐coated sensors, for f‐MVPIKI but not for f‐MEQQNK. In summary, these data demonstrate that peptide‐coated surfaces combined with graphene can be used as a biomedical tool to distinguish between different cell types.

The observed differences between the peptides are due to the different peptide sequences, which by design differ in properties such as hydrophobicity, charge, the presence, and position of certain amino acids. In addition, cell morphology and size may also play a role. The observation that peptide‐coated graphene sensors can distinguish between two different cell types suggests that these interfaces have great potential for applications in biomedical research and diagnostics. The peptide sequences can be modified further to achieve the desired interaction with the cells of interest.

In all cases, the addition of graphene significantly increased the signal‐to‐noise ratio, allowing differentiation of THP1 and HL60 cells using f‐MVPIKI peptides, which was not possible with standard gold chips. These results clearly demonstrate that graphene plays a critical role in enhancing the sensing performance of plasmonic biosensors. To put this into context, we have summarized the signal enhancement observed with graphene in Table 1 and compared it with previous work. As our study specifically focuses on an SPR configuration with a monolayer of graphene deposited on a gold chip, we have limited our comparison to similar configurations in the literature.^[^ ^19^, ^24^ ^]^ Of the two other works we found, one observed a 37% enhancement with streptavidin and the other observed an 80% enhancement with an antibody (cholera toxin).

Our work clearly demonstrates that much higher signal enhancements for monolayer graphene, up to 600%, can be achieved experimentally. We also show that this enhancement is general and can be applied to several biologically relevant systems, such as antibody‐virus and peptide‐cell interactions. This signal enhancement can be attributed to several factors: the ability of graphene to enhance the plasmonic field and interact with the evanescent wave due to its high conductivity,^[^ ^19^, ^31^ ^]^ the higher refractive index sensitivity with graphene^[^ ^17^, ^20^, ^31^ ^]^ (see Figure S3, Supporting Information), the high adsorption capacity of graphene^[^ ^17^ ^]^ for biomolecules and the better ligand immobilization due to π–π interactions on the graphene surface.^[^ ^24^ ^]^

## 3 Conclusion

In summary, adding a monolayer of graphene to the gold surface of SPR chips significantly enhances signals by up to 600%, which is much greater than previously reported. This study is the first to demonstrate that this enhancement can be applied to various biologically relevant systems, including antibody‐virus and peptide‐cell interactions. The improved signal‐to‐noise ratio also enabled the distinction of different cell types, which was not possible with standard gold chips. The signal enhancement with graphene is attributed to improved plasmonic interactions and the immobilization of molecules on the graphene surface. This versatile, adaptable graphene‐enhanced plasmonic interface can be used to study diverse biochemical interactions in real time, providing a powerful tool for potential applications in biomedical research, diagnostics, and therapy. Future studies will investigate the scalability, reproducibility, and long‐term stability of this platform under different operating conditions. Additionally, the research will examine the intricate interactions between small peptides and specific cell surface markers relevant to disease diagnostics. The current study's promising results support using monolayer graphene as a robust sensing layer for surface plasmon resonance (SPR)‐based biosensing. This has the potential to easily translate to other point‐of‐care electrical biosensors,^[^ ^60^ ^]^ such as graphene field‐effect transistors.^[^ ^30^, ^61^ ^]^ Graphene's enhanced sensitivity and selectivity also open the door to biosensing in complex biological fluids, where matrix effects can contribute to nonspecific binding.

## 4 Experimental Section

### Materials

4‐mercaptobutyric acid, 1‐Pyrenebutyric acid, N‐Hydroxysuccinimide (NHS), 1‐Ethyl‐3‐(3‐dimethylaminopropyl) carbodiimide (EDC), Bovine Serum Albumin (BSA) solution, and Ethanolamine were from Sigma–Aldrich. Monolayer Graphene on Polymer Film was purchased from Graphenea. All chemicals utilized in the experiments were of analytical grade or higher quality. C1 assay buffer (130 mM NaCl, 10 mM HEPES, 5 mM KCl, 2 mM CaCl2, 5 mM glucose, pH 7.4; Carl Roth). Antibodies A20 (Cat. No. 690055), A20R (Cat. No. 690298) and A20‐h1 (Cat. No. 692379), and AAV2 empty capsids (Cat. No. 66V020) were provided by PROGEN.

### Multiparametric SPR (MP‐SPR)

The SPR experiments were conducted using an MP‐SPR device (MP‐SPR Navi 200, BioNavis Ltd., Tampere, Finland) equipped with gold chips (BioNavis Ltd., Finland) and a flow cell with a volume of 1µl channel^−1^. The sensor slides, constructed of glass, were coated with a 2 nm layer of chromium and 50 nm layer of gold. SPR measurements were conducted in angular scan mode to monitor real‐time changes in the position of the minimum SPR peak. The biomolecular interactions were analyzed at a wavelength of 670 nm. Before starting the SPR experiments, the sample injection system and flow channels were carefully washed and filled with a running buffer solution. Throughout the duration of the SPR measurement, the flow rate varied according to the step of the binding experiment, and the sensor temperature was maintained at 22 °C.

### Graphene Enhanced SPR Chip Preparation

The monolayer graphene sheet was transferred onto the SPR chips using a standard wet transfer method.^[^ ^60^, ^62^, ^63^ ^]^ This monolayer graphene contained a thin layer of PMMA polymer film. The PMMA/graphene/gold chip was then air‐dried for 30 min. Afterwards, it was cured at 150 °C for one hour and left under vacuum at room temperature overnight. By doing so, the PMMA/graphene layer was fully in contact with the SPR chip. Now, to remove the PMMA layer, the chip was immersed in acetone and kept in the oven at 50 °C for one hour. After this, the chip was immersed in isopropanol for one hour at room temperature. Now the chip was washed with acetone and blow‐dried using nitrogen. The graphene‐coated chips were characterized using atomic force microscopy (AFM) and Raman spectroscopy (Figure S2, Supporting Information). These results confirm the presence of a high‐quality graphene layer. Furthermore, scanning electron microscopy (SEM) was performed on graphene‐coated gold substrates both before and after the SPR experiments. This confirmed that the monolayer graphene remained intact and structurally stable throughout the sensing process (see Figure S6, Supporting Information).

### Functionalization of Gold and Graphene‐Enhanced SPR Chips

The ligand (peptide or antibody) was immobilized onto both gold and graphene‐enhanced sensor surfaces using amine‐coupling chemistry. For the gold chip, the bare sensor surface was first cleaned using oxygen plasma for 10 min, rinsed with ultrapure water, and dried under a nitrogen stream. The chip was then immersed in 4‐mercaptobutyric acid for 60 min to functionalize the surface. In the case of the graphene‐coated chip, the sensor surface was rinsed with ultrapure water, dried under a nitrogen stream, and then incubated in 1‐pyrenebutyric acid for 60 min.

After these respective functionalization steps the SPR chips were placed in the slide holders for the remaining experiments. The complete sensogram having various parts of the whole experiment, is available in (Figure S1, Supporting Information). An initial baseline was recorded in the running buffer, followed by surface activation through a 7‐min injection of a 1:1 mixture of 0.1 M NHS and 0.4 M EDC at a flow rate of 20 µl min^−1^. The ligand peptide (1 mM) or antibody (8 µg ml^−1^) was immobilized onto the activated surfaces of both chips. To block any unreacted esters, a 7‐min injection of 1 M ethanolamine hydrochloride (pH 8.5) was applied. Finally, analytes such as living cells (275k cells ml^−1^) or viruses (10 pM) were flowed over the sensor surfaces at a constant flow rate for detection.

### Cell Culture

HL‐60 and THP‐1 cells (CLS) were cultured in RPMI‐1640 media (Biowest) supplemented with fetal calf serum (10%; Sigma Aldrich), penicillin‐streptomycin (1 unit ml^−1^; Biowest), and L‐glutamine (2 mM; Biowest) in cell culture suspension flasks. Cells were incubated at 37 °C and 5% CO2 and were adjusted three times per week to a cell number of 300.000/ml to keep a suitable confluency. For experiments, cells were taken from cell culture suspension, were centrifuged (300 g; 10 min), and subsequently resuspended to a cell number of 275.000/ml in C1 assay buffer (130 mM NaCl, 10 mM HEPES, 5 mM KCl, 2 mM CaCl2, 5 mM glucose, pH 7.4; Carl Roth). Cells were stored on ice until the start of the experiment to keep their viability.

### MP‐SPR Data Analysis

The MP‐SPR Navi DataViewer software and TraceDrawer ease the extraction and analysis of data obtained from MP‐SPR measurements, including the capture of cells on a peptide‐modified sensor. After the completion of measurements, the software allows users to access and analyze the captured data. Theoretical analysis was done using LayerSolver software in simulation mode.

### Statistical Analysis

All statistical analyses were carried out using BioRender R statistical software (version 4.2.2).

For the analysis presented in Figure 2, the Shapiro‐Wilk test was used to evaluate whether the data distributions for the “Gold” and “Graphene” groups significantly deviated from normality. To assess the equality of variances between the two groups, Levene's test was performed. Subsequently, an independent samples *t*‐test, executed as a two‐tailed test, was employed to compare the means of the “Gold” and “Graphene” groups. The objective of this analysis was to ascertain whether there was a statistically significant difference in either direction. Furthermore, Cohen's d was calculated to measure the effect size, providing a standardized indication of the magnitude of the difference between the group means.

Two‐way analyses of variance (ANOVA) were employed to evaluate the effects of multiple independent variables on the dependent variable, namely the surface plasmon resonance (SPR) signal. As illustrated in Figure 3, the analysis evaluated the impact of surface type (Gold vs. Graphene) and virus serotype (AAV2 vs. AAV8), thereby representing the interaction of A20, A20‐h1, or A20R antibodies with each virus. As illustrated in Figure 4, the analysis investigated the impact of peptide sequence (f‐MVPIKI and f‐MEQQNK) and surface type (Gold and Graphene). Statistical analysis in Figure 5, the independent variables were two peptides (f‐MVPIKI and f‐MEQQNK) and cell types (THP1 and HL60). In all cases, the SPR signal was utilized as the dependent variable. For each analysis, statistically significant main effects and interaction effects were followed by post hoc pairwise comparisons using Bonferroni correction to adjust for multiple comparisons. The objective of these tests was to evaluate the differences between levels of one factor within each level of the other. The magnitude of the effects was calculated using partial eta‐squared, which indicates the proportion of variance explained by each independent variable and their interaction. The statistical significance of each test was determined by the *p*‐value, with a threshold of *p* < 0.05 being considered significant.

## Conflict of Interest

The authors declare no conflict of interest.

## Supporting information



Supporting Information

## Data Availability

The data that support the findings of this study are available from the corresponding author upon reasonable request.
