# Supplementary material for: Graphene‐Enhanced Plasmonic Interfaces: A General Strategy for Highly Sensitive Detection of Biomolecular Interactions
Source: Adv Healthc Mater. 2025 Sep 4;14(31):e01723. doi: 10.1002/adhm.202501723 (PMC12683200; doi:10.1002/adhm.202501723)
Supplement: Supplementary file 1 — Supporting Information [file ADHM-14-0-s001.docx]

**Supplementary data**

**Graphene-Enhanced Plasmonic Interfaces: A General Strategy for Highly Sensitive Detection of Biomolecular Interactions**

Ahmar Hasnain, Heiko Heilmann, Nghi Luong Phuong Le, Klaus-Ingmar Pfrepper, Andreas Wruck, Peter Groß, Bernd Bufe, Alexey Tarasov^*^

A. Hasnain, H. Heilmann, N.L.P. Le, B. Bufe, A. Tarasov

Faculty of Computer Sciences and Microsystems Technology, Kaiserslautern University of Applied Sciences, Amerikastr. 1, 66482 Zweibrücken, Germany

E-mail: [alexey.tarasov@hs-kl.de](mailto:alexey.tarasov@hs-kl.de)

A. Wruck, P. Groß

Faculty of Applied Logistics and Polymer Sciences, Kaiserslautern University of Applied Sciences, Carl-Schurz-Str. 10-16, 66953 Pirmasens, Germany

K-I. Pfrepper

PROGEN Biotechnik GmbH, Maaßstraße 30, 69123 Heidelberg, Germany

1. **SPR sensogram**

**Figure S1** shows a detailed surface plasmon resonance (SPR) sensogram. It illustrates the multistep procedure of the assay conducted on graphene-coated SPR sensors. The experiment begins with an incubation period at room temperature with a linker molecule. Next, the sensor is inserted into the SPR measuring chamber and the measurement procedure begins. The sensogram shows that the assay comprises several steps, beginning with an initial baseline of running buffer. Next, a mixture of EDC/NHS is injected into the fluidic chamber. Two sensograms represent the dual-channel function. One channel is used as the target assay and the other as the control assay.

Following the activation of linker molecules by EDC/NHS, a new baseline is established. Then, ligand molecules are injected into the chamber through both channels. After the ligand (in this case a peptide) has been immobilised, any unreacted esters are blocked with ethanolamine. Following this step, the baseline is re-established once more. Then, the analytes are injected into both channels. In this configuration, the target channel contains a single analyte. The second channel contains an alternative analyte, thereby serving as the control experiment.

**
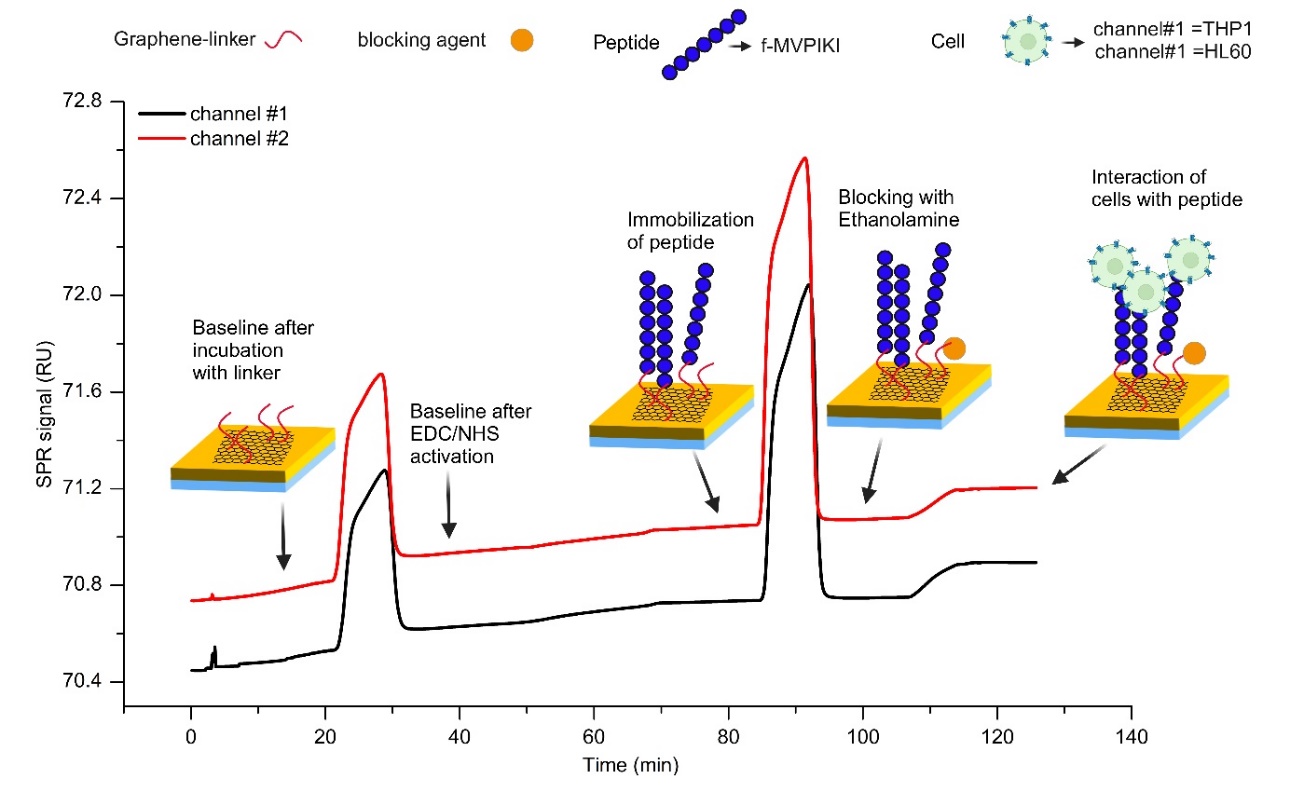
**

**Figure S1.** A sensogram showing the full SPR assay procedure conducted on graphene, aimed at examining the interactions between THP1 and HL60 cells with f-MVPIKI peptide.

This experiment can also be transferred to other assays. Some experiments were conducted on a gold surface using a plain SPR gold chip. The same chemistry was followed for the antibody-virus assay. The antibody was used as the ligand instead of the peptide, and the virus particles were used as the analyte instead of the cells.

1. **Graphene coated SPR sensor chip**

**Figure S2**a presents Surface Plasmon Resonance (SPR) reflectance curves for two sensor setups: bare gold (Au) and graphene-coated gold (Gr-Au). Reflectance is plotted against the SPR angle at three wavelengths-670 nm (solid), 785 nm (dashed), and 980 nm (dash-dot). For the bare gold surface (blue), SPR dips shift to higher angles and become deeper as the wavelength increases, reflecting typical SPR behavior. With the addition of a graphene layer (red), the SPR angles increase further and show changes in dip depth across all wavelengths. This shift indicates a higher effective refractive index near the surface due to graphene, which modifies the surface plasmon conditions. These changes confirm successful graphene functionalization and demonstrate its impact on SPR sensing performance.


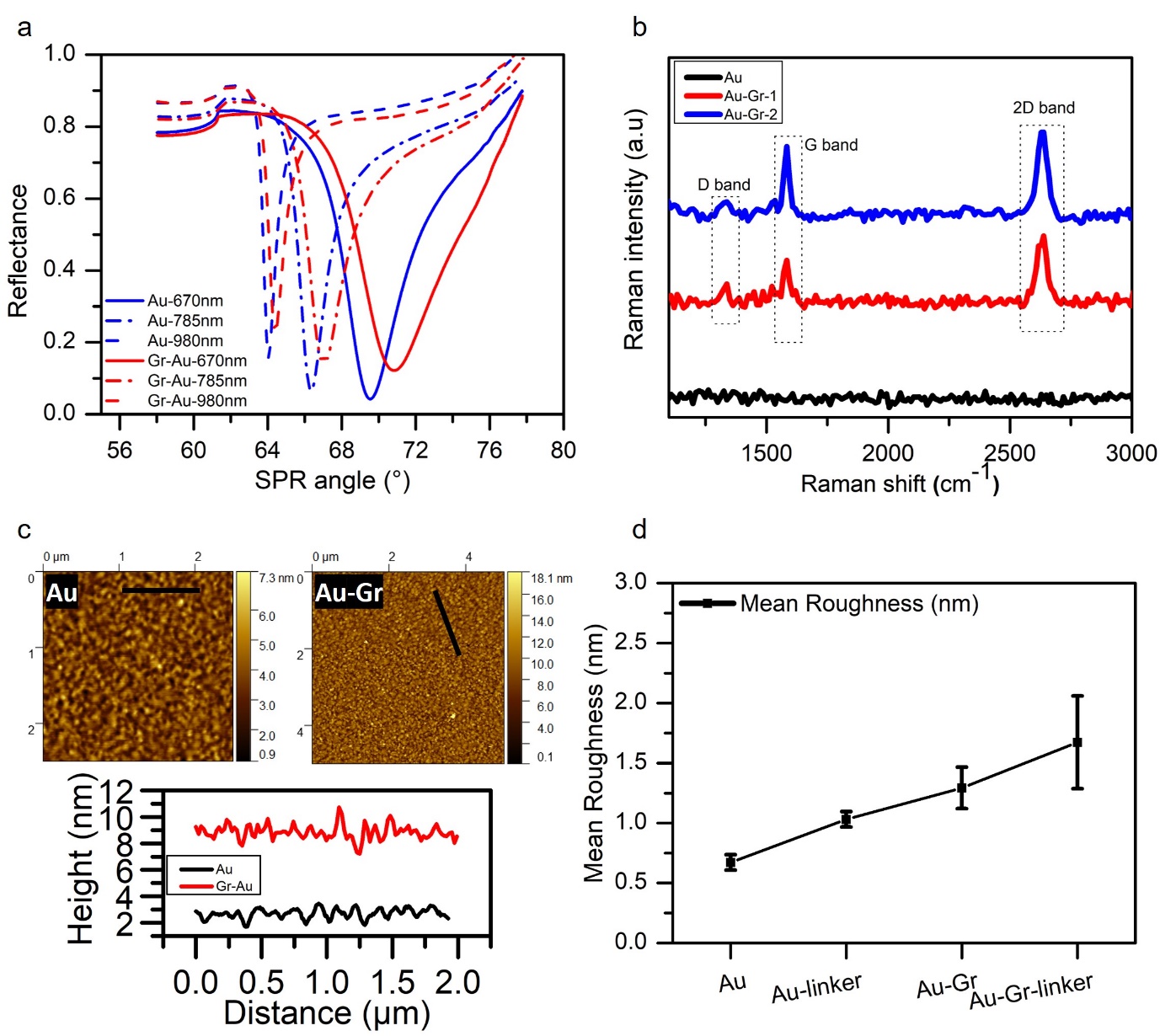


**Figure S2.** (a) Full SPR angular spectra for bare Au (blue) and graphene coated Au (red) from three excitation wavelengths (670 nm, 785 nm, 980 nm). (b) Raman spectra of plain gold SPR chip (black), graphene coated SPR chip (red and blue). (c) SPR surface topography obtained from atomic force microscope (AFM) analysis. Top left: plain gold chip (Au). Top right: graphene coated gold chip (Au-Gr). Bottom: average line profile of plain gold (black) and graphene coated gold chip (red). (d) Mean roughness of the measured area of SPR sensor for bare gold (Au), gold with linker (Au-linker), graphene-coated gold (Au-Gr) and graphene-coated gold with linker (Au-Gr-linker).

Figure S2b shows Raman spectra for bare gold (black trace) and two traces of graphene-coated gold (red &blue). The bare gold spectrum is featureless, as gold is not Raman active in this range. In contrast, both graphene spectrum displays distinct graphene peaks: the G-band, indicating sp² carbon bonding, and the 2D-band, which suggests the presence of monolayer graphene. A weaker D-band, linked to lattice defects, is also visible. The low D-band intensity relative to the G and 2D bands confirms the graphene is of good quality. These results validate the successful and quality deposition of graphene on the gold surface.^[1–3]^ Figure S2c AFM images and height profiles compare the surface topography of bare gold (Au) and graphene-coated gold (Au-Gr). The surface of the gold exhibits a granular texture, which is characteristic of thin gold films. After graphene deposition, it conforms to the underlying gold structure. Different height profiles indicate that graphene slightly modify local surface features. This analysis confirms graphene coverage and provides insight into its effect on surface morphology. Figure S2d shows the average surface roughness measured at two different stages of sensor modification. For the bare gold surface (Au), the roughness increases slightly after the addition of the linker molecule, indicating surface modification. With graphene the roughness increases further. This confirms successful linker functionalization of both sensor surfaces.

1. **Refractive index sensitivity enhancement**

To further explore the effect of monolayer graphene on the refractive index sensitivity of Surface Plasmon Resonance (SPR), we conducted experimental and theoretical analyses comparing a gold film (Au) and a graphene-coated gold system (Au-Gr) using the LayerSolver software in simulation mode based on the Fresnel equations. SPR curves of plain gold(red) and monolayer graphene coated Au chips(blue) are shown in Figure S3a. Deposition of monolayer graphene leads to the angular shift of 1.14°. Solid black lines are fitted curves based on a multilayer model. Using the fitted parameters as summarized in Table S1, we simulated the impact of refractive index (RI) changes in the buffer layer.

Table S1**.** SPR sensor configuration and parameters for fitting.

| Gold | | | | Gold-Graphene | | | |
| --- | --- | --- | --- | --- | --- | --- | --- |
| Layer | **Thickness**  **d(nm)** | **Refractive Index, n** | **Extinction Coefficient, k** | **Layer** | **Thickness**  **d(nm)** | **Refractive Index, n** | **Extinction Coefficient, k** |
| glass | 0.00 | 1.51394 | 0.00000 | **glass** | 0.00 | 1.51394 | 0.00000 |
| Cr | 1.70 | 3.20000 | 2.00000 | **Cr** | 1.70 | 3.20000 | 2.00000 |
| Au | 51.03 | 0.18638 | 3.88471 | **Au** | 51.03 | 0.18638 | 3.88471 |
| buffer | 0.00 | 1.32700 | 0.00000 | **Gr** | 1.24 | 3.00000 | 0.47548 |
|  |  |  |  | **buffer** | 0.00 | 1.32700 | 0.00000 |

The results, presented in Figure S3b and c, show the reflectance curves for Au and Au-Gr systems, respectively. Each curve represents the refractive index change of Δn=0.005.^[4]^ Notably, the addition of monolayer graphene results in a more pronounced shift in the SPR angle compared to bare gold for the same RI changes. At each value of refractive index graphene coated SPR chip gave a higher SPR angle compared to gold chip.

**Figure S3.** (a) SPR scan curves at 670 nm for the bare Au film (red) and the monolayer graphene-coated Au film (blue), obtained when the sensing chamber was filled with buffer. The black solid lines represent the fitted curves. (b) Simulated SPR scan curves from the bare Au film at varying refractive indices. (c) Simulated SPR scan curves from a monolayer of graphene-coated bare Au film at varying refractive indices. (d) Corresponding SPR angle-refractive index plot for the bare Au film (black) and the monolayer graphene-coated Au film (red).

To quantify this effect, we plotted the SPR angle as a function of the refractive index for both systems Figure S3d. The Au-Gr system exhibited a greater sensitivity, with a steeper slope in the SPR angle shift than the Au-only system. The sensitivity (S) was calculated using the simple relationship:^[5]^

$$S=\frac{\Delta\theta}{\Delta n}$$

where Δ*θ* is the shift in SPR angle, and Δ*n* is the refractive index change. The incorporation of monolayer graphene resulted in a sensitivity enhancement of 6.66%. These findings are in accordance with theoretical predictions of approximately 2.5% for monolayer graphene and 25% for ten layers of graphene^[4,5]^. We conclude that the refractive index contributes only minimally to the observed signal enhancement.

1. **Calculation of Detection Limit Signal**

The detection limit (LOD) signal^[6]^ is defined as the minimum detectable signal corresponding to the signal of the reagent blank (injection of running buffer) plus three times its standard deviation. The baseline, which is approximately six to seven minutes in duration, is recorded in the running buffer and was designated as "blank". This allows for the collection of background signal and its inherent variability. The mean signal (*S*_Buffer_) and the standard deviation of the signal noise (*σ*_Buffer_) were calculated for five repetitions with gold and graphene sensors see Figure S4.


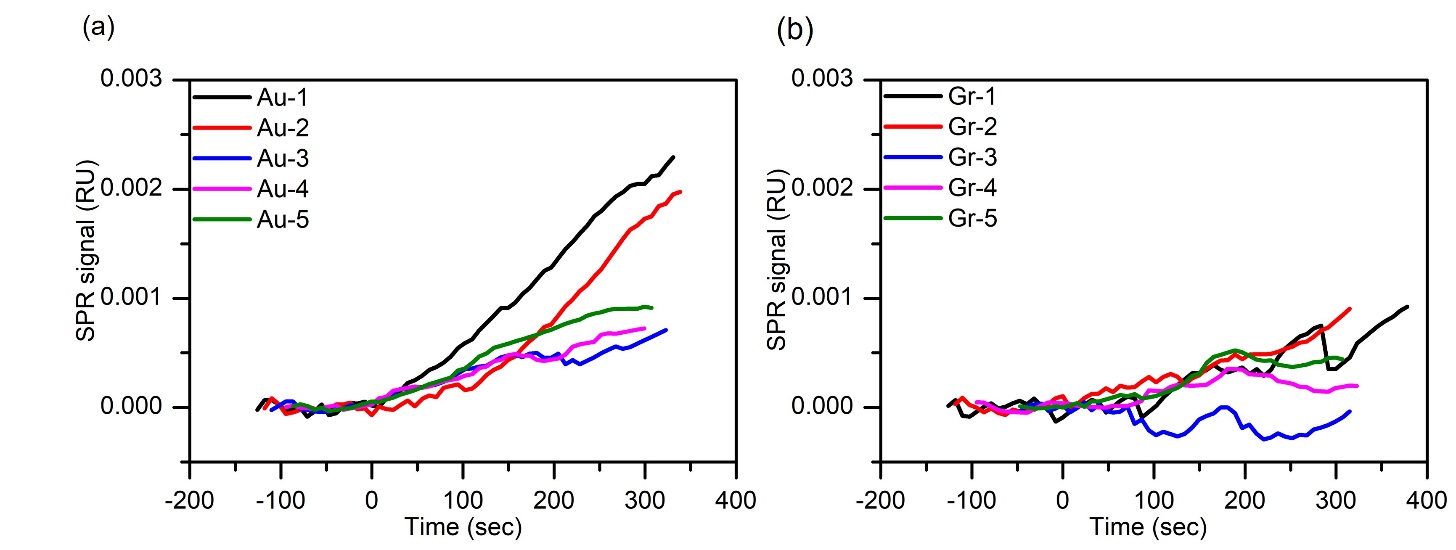


**Figure S4.** (a) SPR sensogram for five independent repetitions in buffer condition (Au-1 to Au-5) using gold sensors. (b) SPR sensogram for five independent repetitions in buffer condition (Gr-1 to Gr-5) using graphene sensors. These data were used to determine detection limit signal and baseline drift.

The final detection limit signal (*S*_dl_​) was computed as:

$$S_{dl}=S_{Buffer}+3\sigma_{Buffer}$$

The resulting S_dl_​ values were used to assess and compare the sensitivity of gold versus graphene surfaces under the same experimental conditions.

1. **Assessment of SPR Sensor Performance in presence of BSA**

In order to assess the impact of a biologically relevant protein matrix on sensor performance, surface plasmon resonance (SPR) experiments were conducted using AAV2 and the A20 antibody in the presence of bovine serum albumin (BSA) at different concentrations (Figure S5). A 35% BSA stock solution was diluted in 10 pM AAV2 virus suspension for this study. As shown in Figure S5 (black bar), AAV2 alone produced a mean SPR response of 0.012±0.001 RU, representing baseline binding to the immobilized A20 antibody. When 0.0035% w/v BSA (≈0.035g/L, 0.526 µM, ~5.26×10⁴ molecules per virus particle) was added to the AAV2 solution, the mean response increased to 0.060±0.010 RU (green bar). To isolate the effect of BSA, 0.0035% BSA in phosphate-buffered saline (PBS) without AAV2 was also tested, yielding a response of 0.036±0.002 RU (red bar), indicating that a significant portion of the increased signal in the green bar was due to nonspecific adsorption of BSA(red bar).^[7]^ Nevertheless, the AAV2+BSA signal remained significantly higher than the BSA-only signal, confirming that specific AAV2-A20 binding still occurred in the presence of BSA.


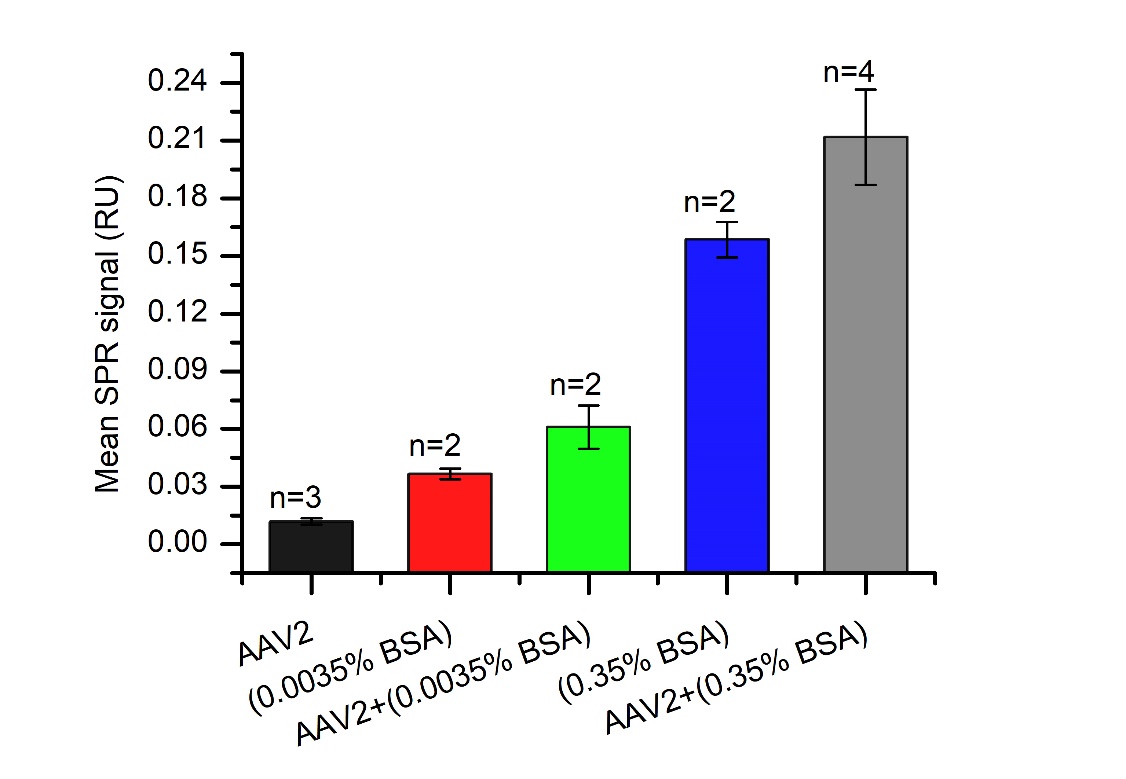


**Figure S5.** Bar charts comparing interactions of A20 antibody functionalized graphene sensor with AAV2 (black), BSA (red and blue), AAV2+BSA (green and grey) based on sensorgrams data. Bars represent mean±SD.

To better approximate physiologically relevant protein conditions, a higher concentration of 0.35% w/v BSA (≈ 3.5 g/L, 52.6 µM, ~5.26×10⁶ molecules per virus particle) was tested with AAV2, resulting in a substantial SPR response of 0.21±0.02 RU (grey bar). The corresponding BSA-only control produced a signal of 0.15±0.009 RU (blue bar). Subtracting the BSA-only signal from the AAV2+BSA signal for each case yielded values higher than those obtained for AAV2 alone, suggesting possible BSA-induced effects such as conformational changes in the viral capsid, increased particle stability, or modulation of antigen-antibody interactions through altered surface accessibility or molecular orientation ^[8–10]^. The large overall signal increase with higher BSA concentrations likely reflects stronger nonspecific adsorption of BSA^[7,11,12]^. Importantly, even at 0.35% BSA, our sensor successfully detected AAV2. These findings demonstrate that the proposed biosensor maintains strong detection performance even under elevated protein background conditions, although further studies are warranted to evaluate its robustness in complex biological media. While undiluted serum can contain 3.5-5g/dL albumin,^[13,14]^ many diagnostic workflows-particularly for SPR-employ sample pretreatment or dilution to reduce matrix effects^[15]^ and minimize nonspecific adsorption^[16–18]^. Our results confirm that the sensor maintains performance at 0.35% BSA background, and we anticipate that with standard dilution of physiologically relevant analytes the method can be extended to higher albumin concentrations typical of raw clinical samples.

To further enhance sensitivity in complex biological matrices, future investigations could adopt advanced surface-blocking strategies. These may include co-immobilizing inert PEG spacers alongside capture ligands to reduce steric hindrance,^[19]^ employing longer PEG-based linker molecules to improve analyte accessibility,^[20]^ using low-concentration BSA as a blocking agent^[21,22]^ and incorporating a small percentage of Tween-20 in the running buffer^[23]^. Such measures are anticipated to suppress nonspecific adsorption, lower baseline noise, and ultimately improve the signal-to-noise ratio when analyzing raw clinical samples.

1. **SEM Analysis of graphene coated SPR chips**

Scanning electron microscopy (SEM) was performed on graphene-coated gold substrates both before and after SPR experiments to evaluate film integrity and assess surface changes resulting from biomolecular interactions. As shown in Figure S6a,c the wet-transferred graphene forms a continuous monolayer with characteristic wrinkles and folding lines,^[24,25]^ and no visible cracks or discontinuities, confirming the successful transfer of high-quality CVD-grown graphene onto the SPR gold chips.


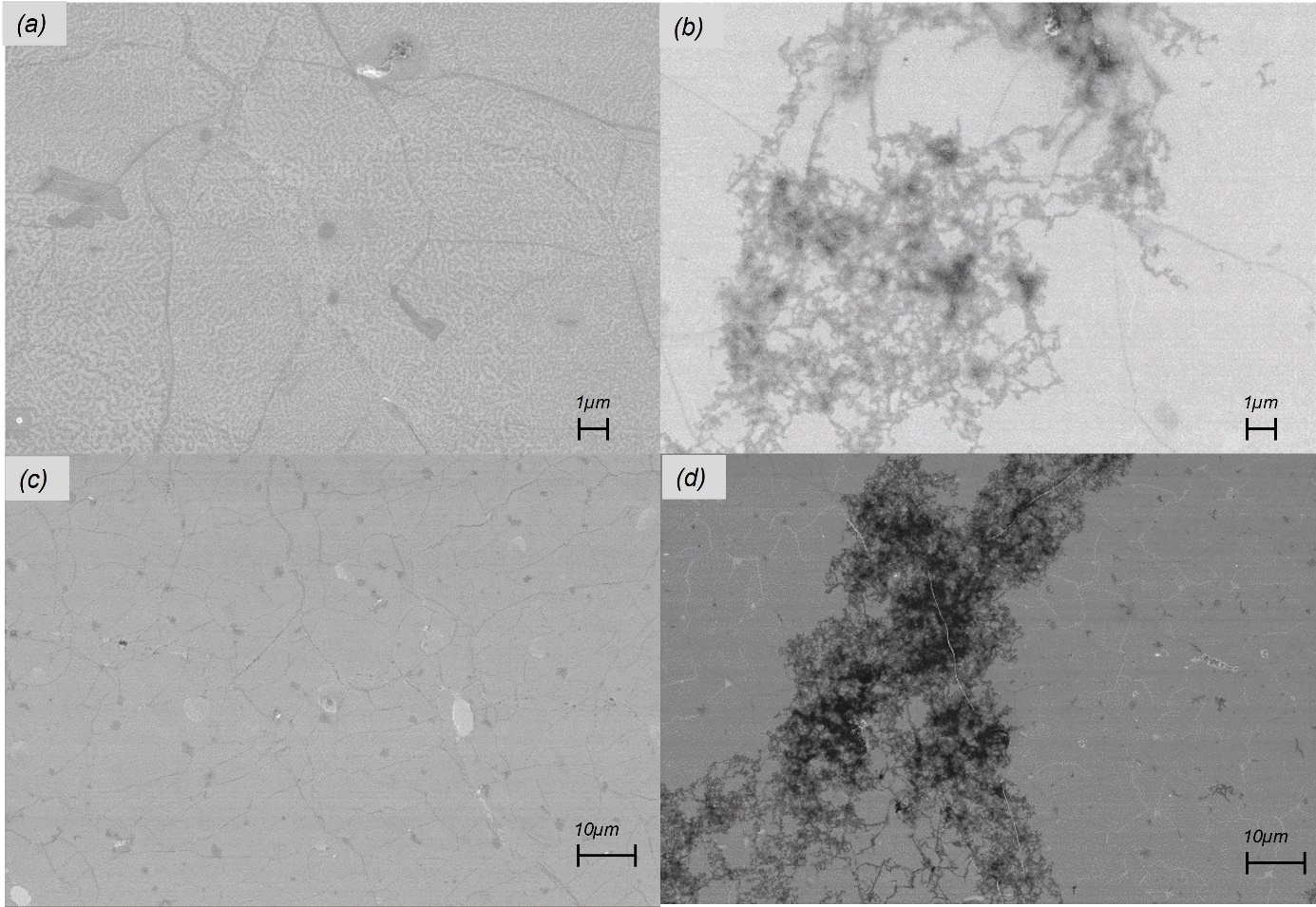


**Figure S6.** SPR surface characterization obtained from scanning electron microscopy (SEM) analysis. (a,c) graphene coated gold chip (Au-Gr). (b,d) graphene coated gold chip after SPR experiment

Following the SPR experiments (Figure S6b,d) the graphene layer remained intact, exhibiting no major signs of delamination or damage. Structural features such as folds and wrinkles persisted, indicating that the film’s morphology remained stable throughout sensor operation.

Notably, darker regions visible in the post-experiment images correspond to areas exposed to antibody and virus solutions. These contrast variations are attributed to biomolecular adsorption during sensing, superimposed on the underlying graphene film, which remains visibly present in the background. This supports both the structural robustness of the graphene and its active role in the biomolecular interaction process.

[1] A. C. Ferrari, J. C. Meyer, V. Scardaci, C. Casiraghi, M. Lazzeri, F. Mauri, S. Piscanec, D. Jiang, K. S. Novoselov, S. Roth, et al., Raman Spectrum of Graphene and Graphene Layers. *Phys Rev Lett* **2006**, *97*, 187401.

[2] A. C. Ferrari, D. M. Basko, Raman spectroscopy as a versatile tool for studying the properties of graphene. *Nat Nanotechnol* **2013**, *8*, 235.

[3] L. M. Malard, M. A. Pimenta, G. Dresselhaus, M. S. Dresselhaus, Raman spectroscopy in graphene. *Phys Rep* **2009**, *473*, 51.

[4] L. Wu, H. S. Chu, W. S. Koh, E. P. Li, Highly sensitive graphene biosensors based on surface plasmon resonance. *Opt. Express* **2010**, *18*, 14395.

[5] T. Tene, J. Svozilík, D. Colcha, Y. Cevallos, P. G. Vinueza-Naranjo, C. Vacacela Gomez, S. Bellucci, The Tunable Parameters of Graphene-Based Biosensors. *Sensors* **2024**, *24*, DOI 10.3390/s24155049.

[6] L. A. Currie, Limits for qualitative detection and quantitative determination.  Application to radiochemistry. *Anal Chem* **1968**, *40*, 586.

[7] J. G. Vilhena, P. Rubio-Pereda, P. Vellosillo, P. A. Serena, R. Pérez, Albumin (BSA) Adsorption over Graphene in Aqueous Environment: Influence of Orientation, Adsorption Protocol, and Solvent Treatment. *Langmuir* **2016**, *32*, 1742.

[8] Y. L. Jeyachandran, J. A. Mielczarski, E. Mielczarski, B. Rai, Efficiency of blocking of non-specific interaction of different proteins by BSA adsorbed on hydrophobic and hydrophilic surfaces. *J Colloid Interface Sci* **2010**, *341*, 136.

[9] Z. Xian, P. Dai, W. Su, D. Xing, C. Sun, H. You, Inhibition of non-specific protein adsorption on PMMA surface: The role of surface modification. *Journal of Saudi Chemical Society* **2023**, *27*, 101755.

[10] Y. Xiao, S. N. Isaacs, Enzyme-linked immunosorbent assay (ELISA) and blocking with bovine serum albumin (BSA)—not all BSAs are alike. *J Immunol Methods* **2012**, *384*, 148.

[11] N. MIURA, D. R. SHANKARAN, T. KAWAGUCHI, K. MATSUMOTO, K. TOKO, High-performance Surface Plasmon Resonance Immunosensors for TNT Detection. *Electrochemistry* **2007**, *75*, 13.

[12] V. Silin, H. Weetall, D. J. Vanderah, SPR Studies of the Nonspecific Adsorption Kinetics of Human IgG and BSA on Gold Surfaces Modified by Self-Assembled Monolayers (SAMs). *J Colloid Interface Sci* **1997**, *185*, 94.

[13] P. Caraceni, M. Domenicali, A. Tovoli, L. Napoli, C. S. Ricci, M. Tufoni, M. Bernardi, Clinical indications for the albumin use: Still a controversial issue. *Eur J Intern Med* **2013**, *24*, 721.

[14] E. Gremese, D. Bruno, V. Varriano, S. Perniola, L. Petricca, G. Ferraccioli, Serum Albumin Levels: A Biomarker to Be Repurposed in Different Disease Settings in Clinical Practice. *J Clin Med* **2023**, *12*, 6017.

[15] J. H. T. Luong, P. Bouvrette, K. B. Male, Developments and applications of biosensors in food analysis. *Trends Biotechnol* **1997**, *15*, 369.

[16] R. L. Rich, D. G. Myszka, Advances in surface plasmon resonance biosensor analysis. *Curr Opin Biotechnol* **2000**, *11*, 54.

[17] J. Homola, Surface Plasmon Resonance Sensors for Detection of Chemical and Biological Species. *Chem Rev* **2008**, *108*, 462.

[18] J. W. Chung, S. D. Kim, R. Bernhardt, J. C. Pyun, Application of SPR biosensor for medical diagnostics of human hepatitis B virus (hHBV). *Sens Actuators B Chem* **2005**, *111–112*, 416.

[19] Ó. Gutiérrez-Sanz, N. M. Andoy, M. S. Filipiak, N. Haustein, A. Tarasov, Direct, Label-Free, and Rapid Transistor-Based Immunodetection in Whole Serum. *ACS Sens* **2017**, *2*, 1278.

[20] N. M. Andoy, M. S. Filipiak, D. Vetter, Ó. Gutiérrez-Sanz, A. Tarasov, Graphene-Based Electronic Immunosensor with Femtomolar Detection Limit in Whole Serum. *Adv Mater Technol* **2018**, *3*, 1800186.

[21] M. Á. García-Chamé, Ó. Gutiérrez-Sanz, E. Ercan-Herbst, N. Haustein, M. S. Filipiak, D. E. Ehrnhöfer, A. Tarasov, A transistor-based label-free immunosensor for rapid detection of tau protein. *Biosens Bioelectron* **2020**, *159*, 112129.

[22] T. Riedel, C. Rodriguez-Emmenegger, A. de los Santos Pereira, A. Bědajánková, P. Jinoch, P. M. Boltovets, E. Brynda, Diagnosis of Epstein–Barr virus infection in clinical serum samples by an SPR biosensor assay. *Biosens Bioelectron* **2014**, *55*, 278.

[23] F. Garay, G. Kisiel, A. Fang, E. Lindner, Surface plasmon resonance aided electrochemical immunosensor for CK-MB determination in undiluted serum samples. *Anal Bioanal Chem* **2010**, *397*, 1873.

[24] M.-A. Yoon, C. Kim, J.-H. Kim, H.-J. Lee, K.-S. Kim, Surface Properties of CVD-Grown Graphene Transferred by Wet and Dry Transfer Processes. *Sensors* **2022**, *22*, DOI 10.3390/s22103944.

[25] X. Yang, H. Peng, Q. Xie, Y. Zhou, Z. Liu, Clean and efficient transfer of CVD-grown graphene by electrochemical etching of metal substrate. *Journal of Electroanalytical Chemistry* **2013**, *688*, 243.
